# The appropriateness of gatekeeping in the provision of reproductive health care for adolescents in Lithuania:the general practice perspective

**DOI:** 10.1186/1471-2296-7-16

**Published:** 2006-03-14

**Authors:** Lina Jaruseviciene, Gwenola Levasseur

**Affiliations:** 1 The Department of Family Medicine, Kaunas University of Medicine, Lithuania; 2 The Department of General Practice, University of Rennes, France; 3 National School of Public Health, Rennes, France

## Abstract

**Background:**

Adolescents' consultation of primary health care services remains problematic despite their accessibility. The reproductive health service seeking behavior of adolescents is the object of much research but little is known about how this behavior is influenced by the gatekeeping system. This study aimed to explore general practitioners' perceptions of the appropriateness of gatekeeping in adolescent reproductive health care.

**Methods:**

Twenty in-depth interviews regarding factors affecting adolescent reproductive health care were carried out on a diverse sample of general practitioners and analyzed using grounded theory.

**Results:**

The analysis identified several factors that shaped GPs' negative attitude to gatekeeping in adolescent reproductive health care. Its appropriateness in this field was questionable due to a lack of willingness on the part of GPs to provide reproductive health services for teenagers, their insufficient training, inadequately equipped surgeries and low perceived support for reproductive health service provision.

**Conclusion:**

Since factors for improving adolescent reproductive health concern not only physicians but also the health system and policy levels, complex measures should be designed to overcome these barriers. Discussion of a flexible model of gatekeeping, encompassing both co-ordination of care provided by GPs and the possibility of patients' self-referral, should be included in the political agenda. Adolescents tend to under-use rather than over-use reproductive health services and every effort should be made to facilitate the accessibility of such services.

## Background

Adolescents' consultation of primary care services remains problematic despite their accessibility [1]. The reproductive health service seeking behavior of adolescents is the object of much research but little is known about how this behavior is influenced by the gatekeeping system.

The gatekeeper, according to Starfield, is the patient's first contact with the health care system, responsible for the provision of primary care as well as for the coordination of care by referring his patients to specialists [2]. Gatekeeping is intended to reduce health expenditures and improve health outcomes [2]. Performed studies, however, demonstrated minimal changes in the consultation of specialists after gatekeeping was replaced by a system with open access to all specialists [3,4]. Moreover, a study by Forrest and al. on child and adolescent referrals to specialty care revealed that gatekeeping arrangements increased such referrals [5]. No evidence was found of a reduction in overall medical care expenditures in the gatekeeping system [6-8], while other research identified negative effects of such a system on patients' confidence and trust in their GP and on their satisfaction with the health care provided [9-11]. Physicians also admitted the negative effects of gatekeeping on the physician-patient relationship [12,13].

The ability of GPs to coordinate medical care efficiently is a crucial prerequisite for the effectiveness of the gatekeeping system [2,6]. Some studies on the delivery of reproductive health services in primary care settings acknowledge general practitioners' efficiency in providing these services for adolescents [14,15]. Many studies, however, emphasize the need for proper training for primary health care providers' and a change of attitude towards adolescents' reproductive health needs [16-19].

Since the adoption of the gatekeeping system in Lithuania in 1997, the GP has been virtually the only health care provider whose services are free of charge and whom adolescents can consult directly when concerned about reproductive and sexual health related issues. During the soviet era, primary care services were segmented by patient age as well as by health problems and general practice did not exist. Training of GPs was introduced in 1992. Two distinct patterns of training were developed for newly graduated and already experienced physicians, some of whom were at pre-retirement age. The current health system framework does not include provision of health services specifically designed for young people.

A survey conducted in Lithuania, however, revealed negative teenage attitudes towards GPs as reproductive health care providers: only 4 % of 13–18 year olds considered GPs as the most appropriate providers [20]. Despite some attempts to study the involvement of GPs in adolescent reproductive health care in Lithuania [21], there is a lack of evidence of the suitability of gatekeeping for such care. The aim of this study, therefore, was to explore general practitioners' experience in adolescent reproductive health care and their perceptions of the appropriateness of gatekeeping.

### Lithuanian health care – the context of transition

Lithuania, the largest of the three Baltic States, re-established its independence from the Soviet Union in 1990. The inherited soviet health system was grounded on the biomedical model, emphasizing technical facilities and underestimating the patient's role. The current legal framework of Lithuania emphasizes the importance of considering the interests of minors in health care provision while the legal age of consent is 18, and for abortion 16 [22,23].

GPs practise in public outpatient departments (polyclinics) and in newly established private clinics. Patients have the right to choose the health care setting as well as the primary health care provider. Direct access, free of charge, to a gynecologist or urologist (traditional provider of reproductive health services for men) is only possible in some private primary care centers that work under contract with Sickness Funds.

Due to expense the plan to provide each GP surgery with gynecological equipment has failed since 10 years. Instead, separate rooms called "room for female examination" or "room for gynecologic examination", were set up in both private and state primary care centers, to be used by all GPs working there. Physicians without this amenity are supposed to use the equipment available in gynecological departments.

Sexual and reproductive health related issues are perceived as a deeply private aspect of the human being in Lithuania. Traditionally, the health sector played a very minor role in the guidance of people towards a healthier sexual life and medical practitioners are still reluctant, however, to move to a more open approach towards sexual and reproductive health issues.

## Methods

This paper reports on one component of a larger project on Adolescent Reproductive Health Promotion Policy in Lithuania. The study was carried out between July and November 2003 in Kaunas, the second city of Lithuania.

According to the Register of National Sickness Funds, primary care services were delivered in Kaunas by 35 private and 46 state practices, in the fall, 2002 [24]. Gender distribution of all GPs running primary health care in Kaunas was not precisely known, but female were predominant – 84.9% of Lithuanian GPs were women [25]. According to the age structure of physicians, 4.9% of Lithuanian GPs were older than 50 years and 2.5% of Lithuanian GPs were older than 60 years 25. It is estimated, that 23% of GPs were trained during the residency in family practice, rest of them retrained mostly from pediatricians and internists [25]. Then, participants were selected in order to achieve diverse representation of views and experiences of general practitioners of differing gender, ages, training and place of work.

The interviews with physicians were held until the saturation of the data. After the 15^th ^interview (11 females and 4 males interviewed) new concepts had not risen and after the 20^th ^interview the collection of data was stopped. The final sample comprised 20 GPs: fifteen females and five males, nine practicing in state primary care settings, eight in private primary care centers, and three practicing in both state and private institutions. Eight of the GPs selected had completed the general practice residency and twelve had become GPs after vocational training (retraining program). The age distribution of participants was as follows: (n = 10) aged 29 – 39, (n = 8) aged 40 – 54 and (n = 2) aged 55 and over.

This study aimed to explore the GPs apprehension of adolescents' sexual and reproductive health issue and their ways of thinking as well as behavior patterns facing the questions emerged. Eventually, the criterion of this qualitative approach was coherence, not representativeness. Individual non-structured in-depth interviews were selected as a method for this study since they provide more privacy exploring personal attitudes towards sexual and reproductive health. All participants were informed of the purpose of the study – exploration of GPs' own experience, thoughts and attitudes towards adolescent reproductive health care. Participants were acquainted with the scheduled publications as well as with the idea of formulating a strategy for the improvement of reproductive health care for adolescents. Confidentiality was warranted to participants.

The principal investigator, a GP, acted as the interviewer. The interviews were held in the offices of participants at the time they themselves had chosen. Physicians were invited to describe actual cases by asking them: "Could you tell me about some of the latest adolescent consultations related to sexual or reproductive health issues?". The interviews were then based on topics revealed by the physicians themselves. The interviews lasted between 50 minutes and 2 hours, mean time – 1 – 1,5 hrs. The interviews were tape recorded, fully transcribed and then analyzed using grounded theory. (Grounded theory could be described as a problem-oriented endeavor in which theories are generated from rich data patterns, elaborated through the construction of plausible models, and justified in terms of their explanatory coherence [26]. This kind of approach is justified for exploration of new areas of research rather for verification of already known phenomena [27]). The data from the interviews were analysed using content analysis to derive themes, generating a primary level interpretation grounded in the data. Each element was coded; codes akin to each other were gathered together as main topics (e.g. gatekeeping, confidentiality, contraception, abortion, etc). These categories formed the interpretative perspective that was revised permanently during the analysis in order to delineate the descriptive model of GPs' views on adolescent reproductive health care. Identified key factors were labeled and illustrated by selected interview quotes. This paper deals only with aspects related to gatekeeping in adolescent reproductive health service provision.

## Results

The analysis identified several factors that shaped GPs' negative attitude to gatekeeping in adolescent reproductive health care. Its appropriateness in this field was questionable due to a lack of willingness on the part of GPs to provide reproductive health services for teenagers, their insufficient training, inadequately equipped surgeries and low perceived support for reproductive health service provision.

### Reluctance to provide sexual and reproductive health services to adolescents

Primary care providers, especially males, consider that conservative social attitudes towards sexuality shape their medical practice and they are ill at ease when dealing with sexual health matters.

*"It's certainly a stupid feeling [*...*] that this is [*...*] domain, well [*...*] not a taboo, but [*...*] some sort of dark and it makes one feel like ashamed*...* and it's awkward to speak about and so on*..."* (GP5M)*.

General practitioners acknowledge that medical guidance is often critical to their young patients – *"I know that these issues are of prime importance in adolescence as well as at an older age" (GP2F)*. However, physicians seem very cautious in discussing sexual and reproductive health matters with their under-age patients. One of the problems is that the duty of providing health care belongs to the parents or legal guardians of a minor. Consequently, parents should be involved in the decision-making process in matters of sexual and reproductive health care. The conflict between the adolescent's reproductive health needs and the parents' preferences presents a dilemma for the physician.

*"These nuances between parents and kids [*...*] are very delicate; moreover, this age (of adolescence) is always so risky; so you are at risk of losing the trust of both of them – parents as well as their kids*..."* (GP7F)*.

The adolescent's demands seem to be the least important component in the triangle physician/teenage patient/parents compared with the legal rights of parents and the professional uncertainty of the physician.

"*I think that *...*if I prescribe contraceptives, let's say, without informing parents [*...*] a lot of misunderstandings could arise [*...*] not on the medical level, I am not talking about side effects, but in relationships*... *It might be that parents are very religious or*... *very conservative and*... *they forbid their daughter to go on a date*... *with some boy*... *I think that I would be*...*I don't know*... *I would become a very bad doctor and I would be*... *stigmatized [*...*] since it (my activity) can be understood*... *as some sort of green light to her." (GP5M)*.

Doctors fear that performing their professional duties may be interpreted as promoting promiscuity or early sexual activity. On the other hand, sexual health seems to be far from the top priorities of GPs. When taking the patient's case history, they usually avoid the reproductive system since it *"isn't so relevant [*...*] as it could cause something bad" (GP9F)*.

Thus, GPs seem to be reluctant to address sexual and reproductive health issues. Social uncertainty around the subject encourages physicians to avoid sexual and reproductive health issues when providing primary care for their adolescent patients.

### Training issues

According to general practitioners, sexual and reproductive health issues were included in the residency program of general practice as well as in the retraining program of practicing physicians. However, participants of the study emphasized their inadequate training in sexual and reproductive health care provision.

General practitioners who completed the full residency program tended to complain that sexual and reproductive health issues during undergraduate as well as postgraduate studies were "*excised" (GP11F), "missed, [*...*] not scheduled [*...*], not emphasized" (GP9F) *topics. Retraining of GPs is much shorter than residency of general practice; still the knowledge obtained during the retraining process tends to be valued more highly.

*"All my life I had been a pediatrician [*...*] and I was happy about these studies [*...*] I studied hard [*...*] I wanted to absorb all lectures [*...*] everything was interesting to me [*...*]. Currently I feel plenitude, I am happy. [*...*] I don't know a lot, that's a tragedy... I perceive deep gaps still I can latch on to some of the problems at least" (GP20F)*.

From the generally positive evaluation of the general practice program it seems that training in sexual and reproductive health was helpful in expanding GPs' personal horizons but it was not consistent enough for their professional needs in reproductive health services. The majority of GPs interviewed are reluctant to discuss these issues, considering that gaps in their knowledge have lead to a lack of competence and of self-confidence.

*"What contraception is the best choice? [*...*] How much is reasonable when decreasing (estrogens), what is permitted after delivery? [*...*] For example, I wouldn't know exactly [*...*]. We don't discuss such questions (with patients)" (GP12F)*.

Added to the lack of specific knowledge is the difficulty of performing gynecological examinations – *"every time I perform a gynecological examination I feel tense" (GP11F)*. Insufficient gynecological examination skills were reported as a major problem to reproductive health service provision by a majority of informants, especially by those completing the vocational training program of general practice.

"...*to perform [*...*] gynecological examination [*...*] I don't know, I haven't the skills [*...*] I can't... I can't be ready for that morally. What's the use of my gynecologic examination if I haven't had any experience? So, what about my examination if I don't detect anything... I have no self-confidence..." (GP17F*).

Perceived professional incompetence forces general practitioners to avoid reproductive health care issues. They are unwilling to tackle this problem area. So, when faced with a reproductive health problem, the most frequent strategy is referral to a gynecologist.

*"Sometimes I get nervous when [*...*] I explain everything, everything is clear, still the patient wants to consult somebody else (a specialist) [*...*]. But when he wants to gynecologist... I let him go... It is better that he would be seen by a gynecologist if there is something wrong [*...*]. When I don't feel confident then [*...*] it's better to let the patient go to the specialist"(GP13F)*.

Consequently, the professional incompetence of general practitioners in reproductive health matters seems to be one of the key explanations for the ineffective gatekeeping in this field.

### Lack of equipment for reproductive health care provision

One critical aspect of the delivery of reproductive health care would seem to be primary care providers' access to gynecological equipment; an overwhelming majority of GPs are still unable to perform gynecologic examinations in their surgeries. "Geographic" isolation of the gynecologic consulting rooms, according to the physicians, is linked to several drawbacks. General practitioners are always short of time and conducting the consultation and examination in two different rooms is especially time consuming. Moreover, various organizational constraints make it even longer.

"...*this room isn't always open [*...*]. Midwives (who practice in this room), well, they are usually there a few hours per day [*...*]. Of course, it's possible to find the key [*...*], but usually you lack time" (GP15M)*.

According to GPs, adolescents can be especially disturbed by this requirement since the privacy of consultation could be violated. The patient's move from the surgery to the place named "room for gynecologic examination" can be easily observed by other patients waiting to see the doctor. As primary care facilities are usually located in comparatively small communities, it is likely that there will be someone in the waiting room who knows the girl. Consequently, the reason of her consultation can be easily disclosed. In the same way, using the equipment in gynecological departments is and embarrassing for the teenage patient and is not really a feasible alternative because of gynecologists' negative attitude towards GPs.

*"We can't go to gynecologists since they accept us extremely unkindly as [*...*] we would be completely clueless" (GP15M)*.

### Perceived support for reproductive health service provision

The integration of new services into routine primary health care requires a great effort from health care providers as well as support from the environment. General practitioners' perception of the support, or rather lack of support, for adolescent reproductive health care provision from policy level, patients and colleagues affects their performance in their gatekeeper role.

The lack of well-defined policy on adolescent reproductive health care in general, and on general practitioners' duties in particular, fosters ignorance of their reproductive health needs. GPs tend to avoid these issues, presuming that other specialists will deal with them. In fact, responding to adolescents' reproductive health needs in such circumstances seems to be assumed by general practitioners as a benevolent, charitable mission rather than as an essential responsibility of the primary health care provider, acting as "gatekeeper".

"...*nobody provides these services... Well, it might be some lone [*...*] enthusiasts who deal with that [*...*]. Really, I don't know who should do that... It may be [*...*] that physicians, precisely, [*...*] family physicians... should take [*...*] one more load on their back" (GP5M)*.

Prior to the health care reform, reproductive health services were provided by gynecologists and, according to general practitioners, patients still express their preferences to consult gynecologists on reproductive and sexual health problems.

*"Yeah... they do not ask me something in that style [*...*], gynecologist is still in the mind of people [*...*], it might be unusual for them, that family physician [*...*] could talk about this sort of matter" (GP9F)*.

Ambiguity of social attitudes towards adolescent sexual and reproductive health care needs, lack of explicit policy on this issue, self-perceived professional incompetence in reproductive health care and lack of equipment place the GP-gatekeeper in a difficult position. They are reluctant to carry out these duties while their patients are reluctant to assess reproductive health problems with them. It seems that the only support that general practitioners receive in this field comes from their colleagues, other GPs who face the same problems. Although such support is vital psychologically, its impact on the delivery of reproductive health services is somewhat negative.

*"I saw – nobody do this work [*...*] of family physician (do not provide reproductive health services). They (other GPs) said to me "Are you crazy?" (GP7F)*.

## Discussion

The findings of this study suggest that GPs do not feel adequately trained, equipped and supported for adolescent reproductive health care provision. Moreover, primary care providers, being gatekeepers of the health system, expressed reluctance to become highly involved in this field and indicated a preference to refer the under-age patient to a specialist for sexual and reproductive health problems. The study results are relevant to the Lithuanian health care system since they question the suitability of a gatekeeping system in reproductive health care provision for adolescents.

*De jure *reproductive health care services for adolescents are accessible in Lithuania. However, experience shows that sexual and reproductive health issues tend to be avoided by GPs in adolescents' consultations. Moreover, if the reproductive health problem is disclosed during consultation, the general practitioners seem to have a problem identifying serious pathologies that require specialist consultation from simple pathologies. Research data underline the sensitivity of adolescents to any hindrance to their access to reproductive health services [28-31]. The gatekeeper who handles these issues ineffectively may well be seen as a barrier, rather than a facilitator in the health seeking process of adolescent. In conclusion, the accessibility of reproductive health services for adolescents can be considered to be compromised *de facto *because of gatekeeping.

The findings of the study suggesting major problems in adolescent reproductive health care are consistent with abundant research data from other countries indicating GPs' difficulties in delivering sexual and reproductive health services for teenagers [32-35]. The participants of our study voiced considerable concern regarding their lack of knowledge and skills in this field. The need for proper training of primary care providers is widely emphasized as an urgent issue for a better response to young people's needs [36-38]. Since it is doubtful whether GPs' perception of their knowledge in areas of common practice adequately reflects their actual knowledge, Tracey argues that the self-directed learning activities of primary health care providers may be misguided [38]. The development of a training curriculum tailored to the needs of health care providers and its integration into the postgraduate training of GPs and into the framework of continuing medical education would seem to meet the demands expressed by general practitioners the best [39,40]. The training programs of GPs should certainly address the issue of gender-specific attitudes, perceptions, beliefs and practices. Regardless the overall conservative attitudes towards sexual and reproductive health practice, the data of this study give a suggestion of specific difficulties met by male physicians in reproductive health care. Existing evidence indicates that educational interventions in adolescent health care specifically designed for GPs is an effective way to achieve improvements in knowledge and skills as well as in self-perceived competence [40].

Although the professional competence of general practitioners is essential in improving adolescent reproductive health care, structural changes aimed at meeting GPs' basic equipment needs are also important. A previous study conducted in Lithuania highlighted the equipment for gynaecological examination as the major factor shaping physicians' activity in adolescent reproductive health care – only 17.2% of Lithuanian GP's are able to use gynaecological equipment in their practices [41]. Data from other countries confirms that physicians' participation in family planning is consistent with the feasibility of providing gynaecological examination [42]. In addition to relatively poor GPs access to gynaecological equipment study revealed a need for the development of a policy including the explicit role of the primary care provider. Political involvement seems to be relevant in stimulating GPs' interest in this field as well as wider patient recognition of GPs as reproductive health care providers. Moreover, an adolescent reproductive health promotion strategy should play a part in the elaboration of incentives that are shown to be effective in the provision of preventive services [43].

These conclusions are the first attempt to address the crucial question of the suitability of gatekeeping in adolescent reproductive health care in Lithuania. This study aimed to explore GPs' perceptions of the appropriateness of such gatekeeping and did not intend to provide a comprehensive evaluation of the effect of gatekeeping on adolescents' reproductive health seeking behaviour or on the health outcomes. A relatively small sample of suitable general practitioners rather than a random sample was appropriate to the needs of this exploratory study. The selection of GPs was performed according to demographic criteria, the format of training perceived in general practice and type of working place (polyclinics and private practices). The sex ratio in sample was set down considering gender distribution of GPs in Lithuania. Two age groups prevailed in final sample: 29 – 39 years ("young" physicians mostly after residence in family medicine) and 40 – 54 years (middle age physicians after re- training in family medicine). Notwithstanding the conformity of the participants to the age, gender structure, training experience of GPs practicing in the primary health care small sample size restricted the possibility of generalisation of the findings. Moreover, the views and experiences of participants may not have been representative of those of the wider general practice community since the study included GPs practicing in one town, the views of physicians from another and, especially, rural areas may have been different on this issue.

Acknowledging the limitations of qualitative study this approach was estimated as favourable for exploration the question of appropriateness of gatekeeping in adolescents' reproductive health care in Lithuania. Previous research on this topic in other countries had already suggested a need for proper training of GPs and a change of their attitude towards adolescents' reproductive health needs [16-19]. However, lack of evidence from Lithuania and specificity of Lithuanian context where gatekeeping duties were allocated to relatively inexperienced GPs encouraged deeper assessment of gatekeeping reality perceived by physicians. As Chalmers et al. [44] noted, the results of a qualitative research project can only be interpreted in a reliable manner if they are studied in a systematic way, together with the results of other studies serving the same research question. Eventually, existing data from other countries and lack of evidence from peculiar Lithuanian context where gatekeeping duties has been allocated to relatively inexperienced GPs grounded the need for qualitative assessment of gatekeeping reality perceived by physicians.

Though, future research should test and prove the findings of this study looking at the broader context and conclusions should be triangulated with a quantitative approach. Studies are required to assess the appropriateness of gatekeeping from the adolescents' point of view. An estimation of the economic impact of gatekeeping in adolescent reproductive health care would be helpful in designing health services for adolescents in Lithuania.

Although the conclusions are tentative, our findings are still sufficient to begin the wider reassessment of gatekeeping in adolescent reproductive health care in Lithuania. GPs' views and experience partly explain the reasons for teenagers' dissatisfaction with GPs as reproductive health care providers, revealed in previous studies [20]. Since factors for improving adolescent reproductive health concern not only physicians but also the health system and policy levels, complex measures should be designed to overcome these barriers.

## Conclusion

General practitioners seem poorly trained with little commitment to the provision of adolescent reproductive health services. Since GPs assume the role of gatekeeper in the Lithuanian health system, adolescents' accessibility to reproductive health services becomes problematic. The various barriers encountered by adolescents in particular mean that gatekeeping restrictions are potentially detrimental to the accessibility of reproductive health care. General practitioners are, however, well placed to play a major role in the provision of these youth services. In developing new reproductive health care patterns for this population, special attention should be paid in designing general practitioner services. A flexible model of gatekeeping should be discussed, encompassing both co-ordination of care provided by GPs as well as the possibility of patients' self-referral [10,45]. Both should be included in the political agenda.

## Competing interests

The author(s) declare that they have no competing interests.

## Authors' contributions

Both authors participated in the design of the study, analyzed the data, and participated in drafting the manuscript. Interviews were carried out by LJ. Both authors read and approved the final manuscript.

## Pre-publication history

The pre-publication history for this paper can be accessed here:



## References

[B1] Jacobson LD, Mellanby AR, Donovan C, Taylor B, Tripp JH (2000). Teenagers' views on general practice consultations and other medical advice. The Adolescent Working Group, RCGP. Family practice.

[B2] Starfield B (1992). Primary care: concept, evaluation, and policy.

[B3] Ferris TG, Chang Y, Blumenthal D, Person SD (2001). Leaving gatekeeping behind – effects of opening access to specialists for adults in a Health Maintenance Organization. N Engl J Med.

[B4] Ferris TG, Chang Y, Perrin JM, Blumenthal D, Person SD (2002). Effects of removing gatekeeping on specialist utilization by children in a Health Maintenance Organization. Arch Pediatr Adolesc Med.

[B5] Forrest CB, Glade GB, Starfield B, Barker AE, Kang M, Reid RJ (1999). Gatekeeping and referral of children and adolescents to specialty care. Pediatrics.

[B6] Martin DP, Diehr P, Price KF, Richardson WC (1989). Effect of a gatekeeper plan on health services use and charges: a randomised trial. AJPH.

[B7] Blumenthal D, Mort E, Edwards J (1995). The efficacy of primary care for vulnerable population groups. Health Services Research part II.

[B8] Escarce JJ, Kapur K, Joyce GF, Van Vorst KA (2001). Medical care expenditures under gatekeeper and point-of-service arrangements. Health Services Research part I.

[B9] Mechanic D, Schlesinger M (1996). The impact of Managed care on patients' trust in medical care and their physicians. JAMA.

[B10] Grumbach K, Selby JV, Damberg C, Bindman AB, Quesenberry C, Truman A, Uratsu C (1999). Resolving the gatekeeper conundrum: What patients value in primary care and referrals to specialists. JAMA.

[B11] Forrest C, Weiner JP, Fowles J, Vogeli C, Frick KD, Lemke KW, Starfield B (2001). Self-referral in point-of-service health plans. JAMA.

[B12] Taylor RT (1989). Pity the poor gatekeeper: a transatlantic perspective on cost containment in clinical practice. BMJ.

[B13] Halm EA, Causino N, Bliumenthal D (1997). Is gatekeeping better than traditional care? A survey of physcians' attitudes. JAMA.

[B14] Pearson VAH, Owen MR, Philips DR, Pereira Gray DJ, Marshall MN (1995). Family planning services in Devon, UK: awareness, experience and attitudes of pregnant teenagers. The British Journal of Family Planning.

[B15] Jonson C, Madeley JR (1995). Contraceptive care in a general practice. The British Journal of Family Planning.

[B16] Wall D, Hounghton G (1997). Family planning – what needs to be thought? A view from general practice in the West Midlands, UK. The British Journal of Family Planning.

[B17] Hellerstedt W, Smith AE, Shew ML, Resnick MD (2000). Perceived knowledge and training needs in adolescent pregnancy prevention: Results from a multidisciplinary survey. Arch Pediatr Adolesc Med.

[B18] Kraus B, Stronski S, Michaud PA (2003). Training needs in adolescent medicine of practicing doctors: a Swiss national survey of six disciplines. Medical Education.

[B19] Garside R, Ayres R, Owen MR, Pearson VH, Roizen J (2000). General practitioners' attitudes to sexual activity under sixteens. Journal of Royal Society of Medicine.

[B20] Jarusevicienë L, Cmieliauskas A, Valius L, Zaborskis A, Zemaitiene N (1999). A study of adolescents attitudes to reproductive health care (Paaugliu poziuris i reprodukcine sveikatos prieziura). Lietuvos bendrosios praktikos gydytojas.

[B21] Jarusevicienë L, Nadisauskiene RJ, Zaborskis A (2000). Primary health care physicians' possibilities to participate in the adolescents' reproductive health care (Pirmines sveikatos prieziuros gydytoju galimybes vykdyti paaugliu reprodukcines sveikatos prieziura). Lietuvos akuserija ir ginekologija.

[B22] Lithuanian law on the rights of patients and compensations for the damage to their health/ Lietuvos Respublikos pacientu teisiu ir zalos atlyginimo istatymas/ No. I- October 3, 1996, new redaction – January 1, 2005.

[B23] Regulation on the Performance of Abortions (Dël nestumo nutraukimo operacijos atlikimo tvarkos). Decree of Ministry of Health of Lithuania, No 50.

[B24] Balciauskaite D, Izdoniene J, Baubinas H Primary health care services and their reimbursment in Lithuania during 1998–2000 years /Pirmines ambulatorine asmens sveikatos prieziuros paslaugos ir ju apmokejimas Lietuvoje 1998–2002 metais/ Sveikatos drauda (in Lithuanian). http://www.vlk.lt/vlk/old_sd.

[B25] Starkiene L, Smigelskas K, Padaiga Z, Reamy J (2005). The future prospects of Lithuanian family physicians: a 10-year forecasting study. BMC Family Practice.

[B26] Laperriere A, Poupart J (1997). La theorisation ancree (grounded theory): demarche analytique et comparaison avec d'autres approaches apparentees. La recherche qualitative: enjeu epistemologiques et methodologiques.

[B27] Corbin J, Strauss AI (1990). Grounded theory research: procedures, canons and evaluative criteria. Qualitative Sociology.

[B28] Frost JJ (2001). Public or private providers? U.S. women's use of reproductive health services. Family Planning Perspectives.

[B29] Stone N, Ingham R (2003). When and why do young people in the United Kingdom first use sexual health services?. Perspectives on Sexual and Reproductive Health.

[B30] Hock-Long L, Herceg-baron R, Cassidy AM, Whittaker PG (2003). Access to adolescent reproductive health services: financial and structural barriers to care. Perspectives on Sexual and Reproductive Health.

[B31] Millstein SG, Nightingale EO, Petersen AC, Mortimer AM, Hamburg DA (1993). Promoting the healthy development of adolescents. JAMA.

[B32] Haley N, Maheux B, Rivard M, Gervais A (1999). Sexual health risk assessment and counseling in primary care: how involved are general practitioners and obstetrician-gynecologists?. American Journal of Public Health.

[B33] Walker ZAK, Townsend J (1999). The role of general practice in promoting teenage health: a review of the literature. Family Practice.

[B34] Huppert JS, Adams Hillard PJ (2003). Sexually transmitted disease screening in teens. Current Women's health Reports.

[B35] Akinbami LJ, Gandhi H, Cheng TL (2003). Availability of adolescent health services and confidentiality in primary care practices. Pediatrics.

[B36] Baraitser P, Allister A, Bigrigg A (1997). Family planning training in the undergraduate curriculum: a national survey and its implications. The British Journal of Family Planning.

[B37] Middleman A, Binns HJ, Durant RH (1995). Factors affecting pediatric residents' intentions to screen for high risk behaviours. Journal of Adolescent Health.

[B38] Tracey JM, Arroll B, Richmond DE, Barham PM (1997). The validity of general practitioners' self assessment of knowledge: cross sectional study. BMJ.

[B39] Michaud PA, Stronski S, Fonseca H, Macfarlane A (2004). The development and pilot-testing of a training curriculum in adolescent medicine and health. Journal of Adolescent Health.

[B40] Sanci LA, Coffey CMM, Veit FCM, Carr-Cregg M, Patton GC, Day N, Bowes G (2000). Evaluation of the effectiveness of an educational intervention for general practitioners in adolescent health care: randomised controlled trial. BMJ.

[B41] Jaruseviciene L, Zaborskis A (2001). Primary health care physicians activity in adolescents' reproductive health care (Pirmines sveikatos prieziuros gydytoju aktyvumas paaugliu sveikatos prieziuroje). Lietuvos akuserija ir ginekologija.

[B42] Kirkkola AL, Virjo I (1998). Finnish health centre physicians' participation in family planning. Scandinavian Journal of Social Medicine.

[B43] Igra V, Millstein SG (1993). Current status and approaches to improving preventive services for adolescents. JAMA.

[B44] Chalmers I, Hedges L, Cooper H (2002). A brief history of research synthesis. Evaluation and the Health Care Professional.

[B45] Gross R, Tabenkin H, Brammli-Greenberg S (2000). Who needs a gatekeeper? Patients' views of the role of the primary care physician. Family Practice.

